# Ozanimod Reduces Serum Neurofilament Light Chain (NfL) and Glial Fibrillary Acidic Protein (GFAP) and Modulates Innate and Adaptive Immunity in Patients with Low-to-Moderate Activity Relapsing–Remitting Multiple Sclerosis

**DOI:** 10.3390/ijms27114933

**Published:** 2026-05-29

**Authors:** Lucienne Costa-Frossard, Luis Brieva, Daniel Apolinar García-Estévez, Jesús Manuel Martín-Martínez, Gary Álvarez-Bravo, María Rosario Blasco-Quílez, José E. Meca-Lallana, María Carmen Calles-Hernández, Cristina Ramo Tello, Carmen Muñoz-Fernández, David Enrique Barbero, Pablo López-Muñoz, José María Prieto González, Antonio Candeliere-Merlicco, Olga Carmona, Javier Riancho, Nuria Sola-Valls, María Carcelén-Gadea, Laura Borrega, Francisco Gascón-Giménez, David Vilanova, Xavier Pérez, Luisa María Villar

**Affiliations:** 1 Department of Neurology, Hospital Universitario Ramón y Cajal, Red Española de Esclerosis, Múltiple (REEM), Red de Enfermedades Inflamatorias (REI), IRYCIS, Universidad de Alcalá, 28034 Madrid, Spain; lufrossard@yahoo.es; 2 Neuroimmunology Group, Department of Medicine, Department of Neurology, University of Lleida, IRBLleida, Hospital Universitari Arnau de Vilanova, 25198 Lleida, Spain; brievaluis@hotmail.com; 3 Department of Neurology, Complejo Hospitalario Universitario de Orense, 32005 Orense, Spain; daniel.apolinar.garcia.estevez@sergas.es; 4 Department of Neurology, Hospital Miguel Servet, 50009 Zaragoza, Spain; jesmartin58@gmail.com; 5 Neuroimmunology and Multiple Sclerosis Unit, Neurodegeneration and Neuroinflammation Research Group, Girona Biomedical Research Institute (IDIBGI), Dr. Josep Trueta University Hospital, 17001 Girona, Spain; garyalvarez.girona.ics@gencat.cat; 6 Neuroimmunology Unit, Department of Medicine, Department of Neurology, University Autonoma de Madrid IRBLleida, Hospital Universitario Puerta de Hierro, 28222 Madrid, Spain; charoblascoquilez@yahoo.es; 7 Multiple Sclerosis CSUR and Clinical Neuroimmunology Unit, Neurology Department, Virgen de la Arrixaca Clinical University Hospital (IMIB-Arrixaca), NICEM Cathedra-UCAM-San Antonio Catholic University, 30120 Murcia, Spain; pmecal@gmail.com; 8 Department of Neurology, Hospital Son Espases, 07120 Palma de Mallorca, Spain; mcalles22@yahoo.es; 9 Multiple Sclerosis Unit, Department of Neurosciences, Germans Trias i Pujol University Hospital, 08916 Badalona, Spain; cramot@gmail.com; 10 Department of Neurology, Hospital Torrecárdenas, 04009 Almería, Spain; mmunozf@outlook.es; 11 Department of Neurology, Hospital de Guadalajara, 19002 Guadalajara, Spain; debarberoj@gmail.com; 12 Department of Neurology, Hospital Universitario Arnau de Vilanova, 46015 Valencia, Spain; lopez_pabmun@gva.es; 13 Department of Neurology, Complejo Hospitalario Universitario de Santiago, 15706 Santiago de Compostela, Spain; josemaoscar.prieto@usc.es; 14 Department of Neurology, Hospital Rafael Méndez, 30817 Lorca, Spain; candeliereantonio@gmail.com; 15 Department of Neurology, Fundació Salut Empordà, 17600 Girona, Spain; occodina@gmail.com; 16 Department of Medicine and Psychiatry, University of Cantabria, 39005 Santander, Spain; javier.riancho86@gmail.com; 17 Service of Neurology, Hospital General Sierrallana-IDIVAL, 39300 Torrelavega, Spain; 18 Clinical and Epidemiological Neuroscience Group (NeuroÈpia) IRBCatSud, Department of Neurology, Hospital Sant Joan de Reus, 43204 Reus, Spain; nuria.sola@salutsantjoan.cat; 19 Department of Neurology, Hospital General Universitario de Valencia, 46014 Valencia, Spain; mariacarcelengadea@gmail.com; 20 Neurology Unit, Hospital Universitario Fundación Alcorcón, 28922 Alcorcón, Spain; laura.borrega@salud.madrid.org; 21 Department of Neurology, Hospital Clínico Universitario de Valencia, 46010 Valencia, Spain; fgascongimenez@gmail.com; 22 Bristol Myers Squibb, 28050 Madrid, Spain; david.vilanovalarena@bms.com (D.V.); xavier.perez1@bms.com (X.P.); 23 Department of Immunology, Hospital Universitario Ramón y Cajal, Red Española de Esclerosis Múltiple (REEM), Red de Enfermedades Inflamatorias (REI), IRYCIS, Universidad de Alcalá, 28034 Madrid, Spain

**Keywords:** multiple sclerosis, neurofilament light chain, glial fibrillary acidic protein, serum biomarkers, ozanimod, real-world evidence, adaptive immunity, innate immunity, inflammation, cytokines

## Abstract

Relapsing–remitting multiple sclerosis (RRMS) is characterized by neuroaxonal damage, astrogliosis and inflammation. These mechanisms may already be active from early disease stages, underscoring the need for sensitive biomarkers capable of capturing treatment-related biological effects beyond conventional clinical measures. In this multicenter, ambispective, observational real-world study, the longitudinal effects of ozanimod on serum biomarkers were evaluated, during the first year of treatment in patients with low-to-moderate activity RRMS. Serum neurofilament light chain (sNfL), glial fibrillary acidic protein (sGFAP) and cytokines associated with immune activity (IFN-γ, IL-17, IL-6, IL-10, and IL-1β) were quantified at baseline, 6 months, and 12 months using an ultrasensitive single-molecule array (SIMOA). Ozanimod was associated with significant reductions in sNfLs and sGFAP at 12 months. Concomitantly, significant decreases in IFN-γ and IL-1β were observed. IL-17 levels remained unchanged in the overall cohort but decreased in patients with higher baseline IL-17 levels. These findings demonstrate coordinated modulation of biomarkers reflecting neuroaxonal damage, astroglial activation, and inflammatory activity under ozanimod treatment in early RRMS in real-world conditions. These results highlight the biological relevance of early intervention within a therapeutic window of opportunity and support the potential utility of serum biomarkers for monitoring biological treatment effects in clinical practice.

## 1. Introduction

Multiple sclerosis (MS) is a chronic immune-mediated disease of the central nervous system (CNS) characterized by inflammatory demyelination, neuroaxonal injury, and neurodegeneration ultimately leading to long-term disability [1]. Relapsing–remitting multiple sclerosis (RRMS), the most frequent clinical course, is defined by episodic inflammatory activity with partial or complete recovery followed by periods of remission [2]. However, accumulating evidence indicates that tissue injury and disability accrual may occur even during early disease stages through mechanisms that are not always captured by conventional clinical or radiological measures [3].

Early diagnosis and prompt initiation of disease-modifying therapy (DMT) are increasingly recognized as key strategies to limit irreversible tissue injury and delay disability accumulation [4]. However, the expanding therapeutic landscape has reinforced the need for treatment individualization, since conventional clinical and magnetic resonance imaging (MRI) measures may not fully capture ongoing subclinical neuroaxonal or glial injury [5]. This has driven interest in the concept of a therapeutic “window of opportunity,” during which inflammatory and glial biology may remain more plastic and amenable to modulation [3,4]. Fluid biomarkers have therefore emerged as complementary tools to monitor underlying disease biology and treatment response [5,6].

Serum neurofilament light chain (sNfL) is a sensitive marker of neuroaxonal damage that correlates with acute inflammatory activity, new MRI lesion formation, and future disease activity risk [5,6,7]. In contrast, serum glial fibrillary acidic protein (sGFAP) reflects astroglial activation, and has been increasingly associated with progression-related pathology, supporting its potential value for monitoring non-relapse-driven disability accrual [5,8,9]. Together, these biomarkers provide complementary information on neuronal and glial injury occurring during the course of MS.

Beyond structural biomarkers, profiling circulating immune mediators can further contextualize the inflammatory pathways contributing to tissue injury [10,11]. Adaptive immune responses, including T helper (Th)1 and Th17-associated pathways, together with innate immune activation, play central roles in MS pathogenesis and lesion evolution [10,11,12]. Interferon (IFN-γ) is a canonical Th1 cytokine implicated in MS immunopathology, while interleukin-17 (IL-17) reflects Th17 activity [10,12]. Interleukin 1-β (IL-1β) is a key mediator of innate immune activation that amplifies inflammatory signaling through microglial and astrocytic activation contributing to CNS inflammation [11,13,14]. Advances in ultrasensitive analytical platforms, such as single-molecule array (SIMOA) technology now enable reliable quantification of low-abundance cytokines in peripheral blood, supporting more granular assessment of immune dynamics in real-world settings [15].

Ozanimod is an oral, selective sphingosine-1-phosphate receptor (S1PR) modulator targeting S1P_1_ and S1P_5_ [16,17,18]. Its primary mechanism involves functional antagonism of S1P_1_ on lymphocytes, leading to receptor internalization and retention of activated lymphocytes within lymphoid tissues, thereby reducing their recirculation and access to the CNS [16,17,18]. In phase 3 trials (SUNBEAM and RADIANCE), ozanimod demonstrated superior efficacy versus interferon β-1a on clinical and MRI outcomes, with a favorable benefit–risk profile [16,17], and data from the long-term extension in the DAYBREAK open-label follow-up supported sustained disease control over extended exposure [19]. Biomarker analyses from pivotal trials have also shown reductions in plasma NfL under ozanimod treatment, linking biological effect to clinical and radiological outcomes [20]. However, real-world data integrating biomarkers of neuroaxonal, astroglial, and immune signaling during early treatment phases remain limited [19].

In this multicenter real-world study, we aimed to evaluate whether ozanimod treatment is associated with longitudinal changes in serum biomarkers of neuroaxonal damage, astroglial activation and immune activity, during the first year of treatment in patients with low-to-moderate activity. We assessed serum biomarkers of neuroaxonal damage (sNfL), astroglial activation (sGFAP), and immune function through cytokines associated with adaptive (IFN-γ, IL-17, IL-6) and innate (IL-1β, IL-6) immunity, as well as the anti-inflammatory cytokine IL-10. By integrating highly sensitive biomarker and cytokine profiling in a clinically relevant early disease population, this study seeks to provide complementary insight into the biological mechanisms underpinning treatment response and to inform biomarker-guided monitoring strategies in RRMS [5].

## 2. Results

### 2.1. Baseline Characteristics of the Patient Cohort

A total of 55 patients with RRMS were included at baseline. Of these 35 were women (63.6%) and 20 (36.4%) men. The median (interquartile range [IQR]) age at symptom onset was 39.5 (33.0–46.0) years and the median age at diagnosis was 42 (35.0–49.0) years. Most patients were treatment-naïve at the time of ozanimod initiation (50/55; 90.9%), while five patients (9.1%) had received one prior disease-modifying therapy.

Clinical disease activity at baseline was low-to-moderate, with a mean (standard deviation [SD]) number of relapses in the year preceding treatment initiation of 0.60 (SD 0.50) and a median of 1.00 (IQR 0.00–1.00). The mean baseline EDSS score was 1.60 (SD 1.01), with a median of 1.50 (IQR 1.00–2.00) (n = 49). MRI baseline data were available for 61.8% (n = 34) of patients, as MRI assessments were performed according to routine clinical practice at the participating centers. These data showed a mean total lesion number of 37.82 (SD 31.34) and a median of 25.00 (IQR 17.00–54.00). Baseline demographic and clinical characteristics are summarized in Table 1.

### 2.2. Biomarker Profile During Ozanimod Treatment

Longitudinal serum biomarker analyses at baseline, 6 and 12 months were available for 50 patients. Changes over time were evaluated for markers of neuroaxonal damage (sNfL), astroglial activation (sGFAP), and immune function (IL-1β, IFN-γ, IL-17, IL-6, and IL-10).

#### 2.2.1. Ozanimod on Axonal Damage

**Figure 1:**
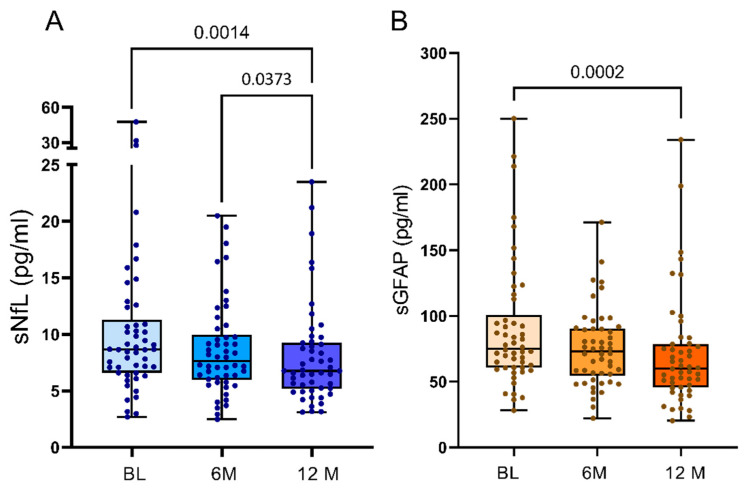
sNfL and sGFAP changes at different time points after ozanimod treatment. (**A**) sNfL changes over time. (**B**) sGFAP changes over time. Values are represented as median (IQR). *p* < 0.05 was considered as statistically significant. BL: baseline; 6M: 6 months; 12M: 12 months.

**Figure 2:**
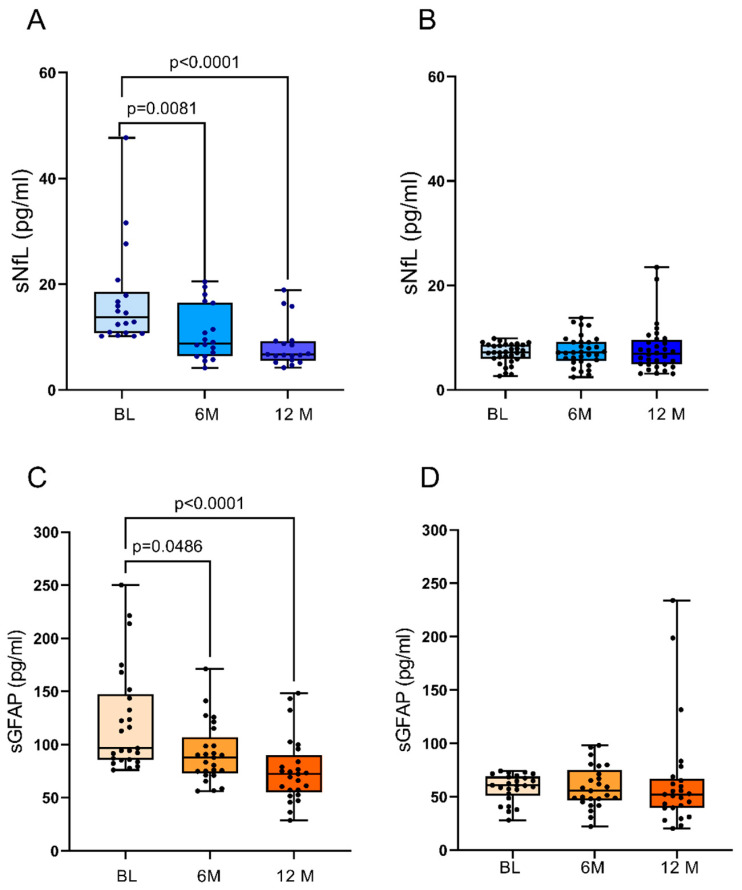
sNfL and sGFAP changes in patients with high vs. low baseline levels after ozanimod treatment. (**A**) Patients with high baseline sNfL levels (n = 18). (**B**) Patients with low baseline levels (n = 32). Stratification was established using a literature-based cut-off value of 10 pg/mL. (**C**) Patients with high baseline sGFAP levels (n = 25). (**D**) Patients with low baseline sGFAP levels (n = 25). Stratification for GFAP was established according to the cohort median value at baseline (75.24 pg/mL). Values are represented as median (IQR). *p* < 0.05 was considered as statistically significant. BL: baseline; 6M: 6 months; 12M: 12 months.

Changes in sNfL were analyzed due to its role as a sensitive biomarker of both acute and chronic axonal damage. After 12 months of ozanimod treatment, median sNfL levels decreased from 8.67 pg/mL at baseline to 6.77 pg/mL at 12 months, corresponding to a 21.9% reduction from baseline (*p* = 0.0014) (Figure 1A). This decline was also evident when compared with 6-month sNfL values (*p* = 0.0373). To assess whether baseline NfL levels influenced the longitudinal response, patients were categorized according to a literature-based threshold of 10 pg/mL [7]. Among patients presenting baseline NfL concentrations above this cut-off (n = 18), a significant reduction was already evident at 6 months compared with baseline (*p* = 0.0081), with median values decreasing from 13.74 pg/mL to 8.84 pg/mL at 6 months, representing a 35.7% reduction, followed by a further and more pronounced decrease at 12 months (*p* < 0.0001), reaching 6.68 pg/mL and corresponding to a 51.4% decrease from baseline (Figure 2A). In contrast, patients with baseline NfL concentrations below the cut-off (n = 32) showed no significant changes over time (Figure 2B).

#### 2.2.2. Ozanimod on Astroglial Activation

We subsequently evaluated sGFAP to investigate the effects of ozanimod treatment in astrocytic activation. Overall, patients exhibited a remarkable decline in sGFAP levels after 12 months of ozanimod treatment compared with baseline (*p* = 0.0002), with median values decreasing from 75.24 pg/mL to 60.17 pg/mL at 12 months, corresponding to a 20.0% reduction from baseline (Figure 1B). Patients were stratified based on baseline concentrations using the cohort median value (75.24 pg/mL) as the cut-off. Patients with higher baseline GFAP levels (n = 25) exhibited a significant decrease at 6 months compared with baseline (*p* = 0.0486), with median values decreasing from 96.67 pg/mL at baseline to 87.93 pg/mL at 6 months, representing a 9.0% reduction, which became more pronounced at 12 months (*p* < 0.0001), reaching 72.36 pg/mL and corresponding to a 25.1% decrease from baseline (Figure 2C). By contrast, GFAP concentrations remained stable over time in patients with baseline values below the median (n = 25), with no significant changes observed throughout the follow-up period (Figure 2D).

#### 2.2.3. Ozanimod Effect on Innate and Adaptive Inflammatory Profile

Immune function was then evaluated by measuring serum levels of different cytokines implicated in the pathological processes of RRMS. Innate immune responses were assessed through IL-1β, whereas adaptive immune activity was examined using IFN-γ and IL-17 levels. IL-6 and IL-10 were considered markers involved in both immune compartments.

**Figure 3:**
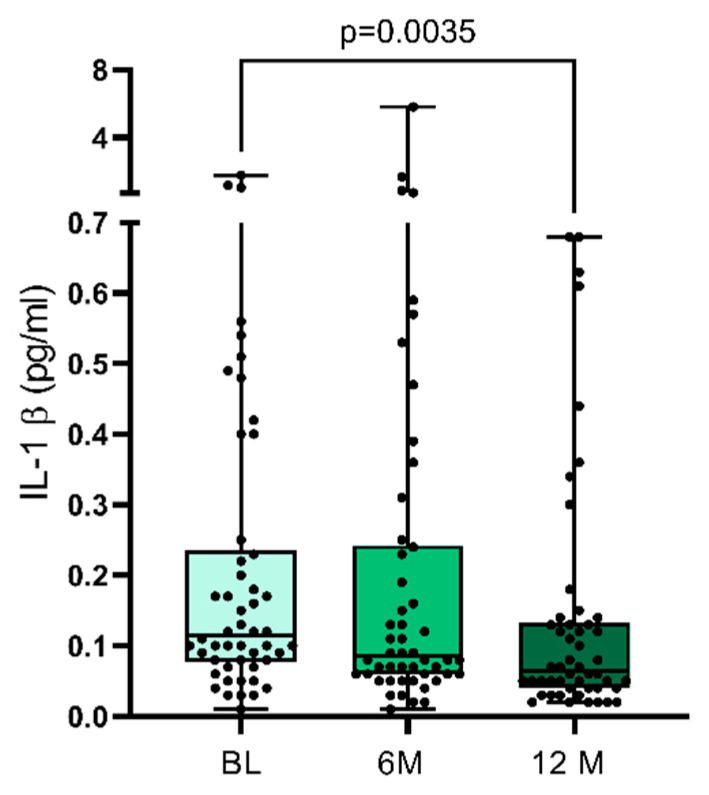
IL-1β changes at different time points after ozanimod treatment. Values are represented as median (IQR). *p* < 0.05 was considered as statistically significant. BL: baseline; 6M: 6 months; 12M: 12 months.

Analysis of IL-1β levels revealed a significant reduction at 12 months of ozanimod treatment compared with baseline in the overall cohort, with median values decreasing from 0.12 pg/mL to 0.07 pg/mL at 12 months, corresponding to a 43.5% reduction from baseline (*p* = 0.0035) (Figure 3).

**Figure 4:**
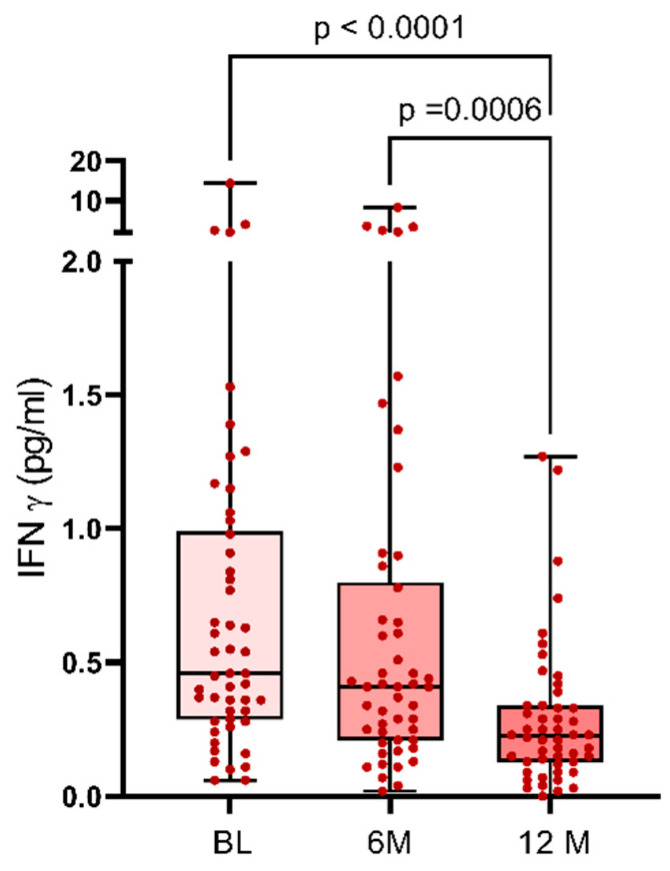
IFN-γ changes at different time points after ozanimod treatment. Values are represented as median (IQR). *p* < 0.05 was considered as statistically significant. BL: baseline; 6M: 6 months; 12M: 12 months.

**Figure 5:**
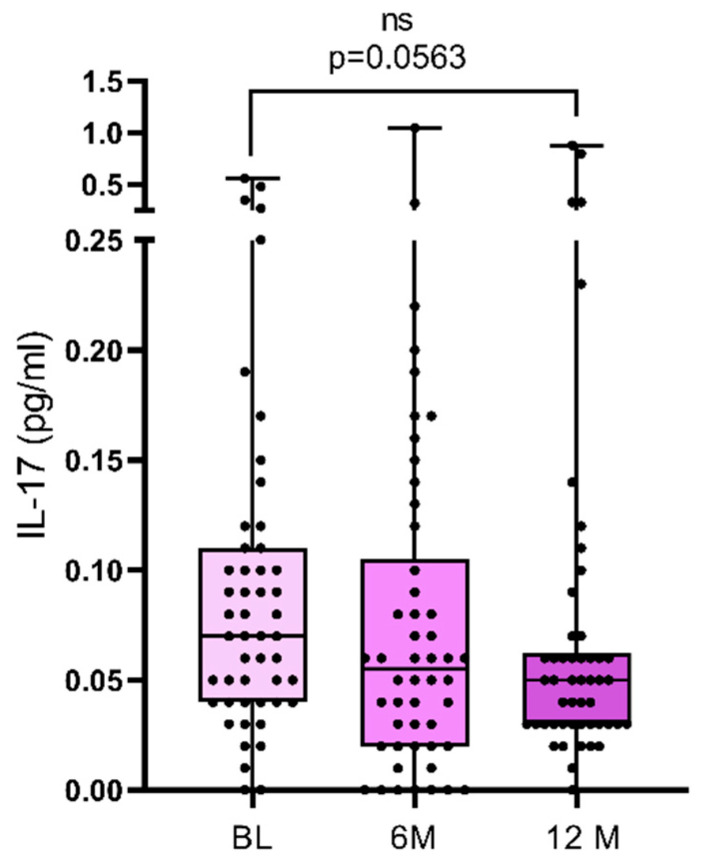
IL-17 changes at different time points after ozanimod treatment. Values are represented as median (IQR). *p* < 0.05 was considered as statistically significant. BL: baseline; 6M: 6 months; 12M: 12 months.

**Figure 6:**
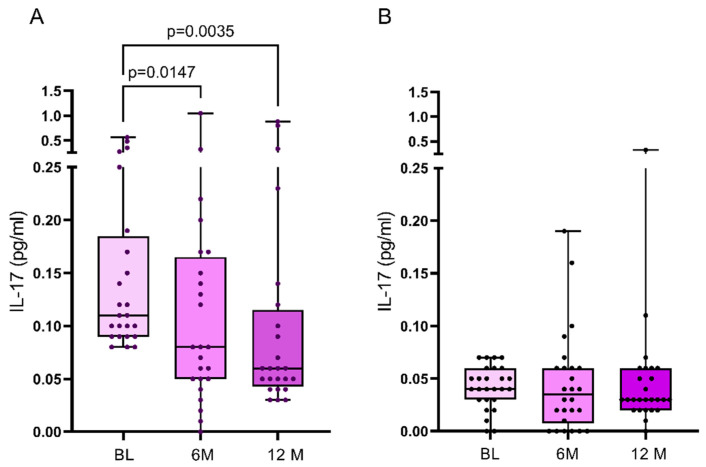
IL-17 changes in patients with high vs. low baseline IL-17 levels after ozanimod treatment. (**A**) Patients with high IL-17 baseline levels (n = 24). (**B**) Patients with low IL-17 baseline levels (n = 26). Stratification was established according to cohort median values at baseline (0.07 pg/mL). Values are represented as median (IQR). *p* < 0.05 indicates statistically significant differences between groups. BL: baseline; 6M: 6 months; 12M: 12 months.

IFN-γ levels decreased after 12 months of treatment compared with both baseline and 6-month measurements (*p* < 0.0001 and *p* = 0.0006; respectively), with median values decreasing from 0.46 pg/mL at baseline to 0.23 pg/mL at 12 months, corresponding to a 51.1% reduction (Figure 4). Nevertheless, IL 17 levels did not change significantly over the follow-up period in the overall cohort but a trend was observed (*p* = 0.0563) (Figure 5). To further explore this finding, patients were stratified according to baseline IL-17 concentrations using the cohort median value (0.07 pg/mL) as the cut-off. In patients with baseline IL-17 levels above the median (n = 24), a significant reduction in IL 17 concentrations was observed at 6 months compared with baseline (*p* = 0.0147), with median values decreasing from 0.11 pg/mL at baseline to 0.08 pg/mL at 6 months, representing a 27.3% reduction, followed by a further and more pronounced decrease at 12 months (*p* = 0.0035), reaching 0.06 pg/mL and corresponding to a 45.5% decrease from baseline (Figure 6A). In contrast, no significant changes were detected in patients with baseline IL 17 levels below the median (n = 26) (Figure 6B).

**Figure 7:**
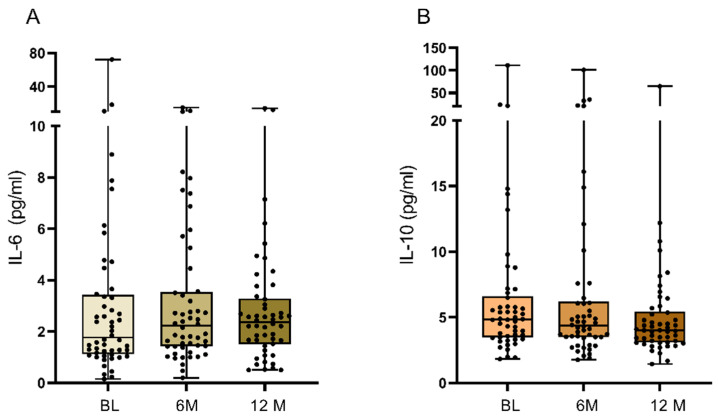
IL-6 and IL-10 changes at different time points after ozanimod treatment. (**A**) IL-6 changes and (**B**) IL-10 changes over time. Values are represented as median (IQR). *p* < 0.05 was considered as statistically significant. BL: baseline; 6M: 6 months; 12M: 12 months.

Serum concentrations of IL 6 and IL 10 remained stable throughout the 12-month follow-up period, with no significant differences observed at either 6 or 12 months compared with baseline (Figure 7).

## 3. Discussion

In this multicenter real-world study of patients, conducted in a well-defined cohort of patients with low-to-moderate activity RRMS, ozanimod treatment was associated with a consistent biological signature across neuroaxonal damage, astroglial activation, and immune function biomarkers over 12 months. Specifically, reductions in sNfL and sGFAP were accompanied by significant decreases in IFN-γ and IL-1β, while IL-6, IL-10, and IL-17 remained stable in the overall population. Taken together, these findings provide a mechanistic framework that aligns with the established clinical efficacy of ozanimod and offers additional insight into its mechanisms of action in a population representative of early and mild disease stages.

The observed decline in sNfL supports a reduction in ongoing neuroaxonal damage, a process closely linked to inflammatory activity, lesion formation, and future disability [21]. This finding is consistent with prior clinical trial data showing that reductions in NfL under ozanimod correlate with lower relapse rates, reduced MRI activity and no evidence of disease activity [20]. In parallel, the significant reduction in sGFAP suggests attenuation of astroglial activation, a process increasingly associated with chronic tissue injury and progression-related biology rather than with acute relapses alone [22]. The combined decrease in sNfL and sGFAP therefore supports the notion that ozanimod may influence both inflammatory and non-acute components of MS pathology, even in patients with relatively low baseline clinical activity. Stratified analyses further refined this interpretation by showing that a relevant proportion of patients presented elevated baseline levels of either sNfL or sGFAP, reflecting underlying subclinical neuroaxonal damage and astroglial activation. In these patients, ozanimod treatment was associated with an early and sustained reduction in both biomarkers, detectable as early as 6 months after treatment initiation.

The interpretation of sGFAP dynamics must be considered in light of disease duration. Increasing evidence suggests that astroglial responses evolve over the course of MS and may become progressively more entrenched over time, thereby limiting their reversibility once chronic CNS inflammatory and neurodegenerative processes are established [23]. In this context, the significant reduction in sGFAP observed in the present study reflects the early disease phase of the included patients, characterized by a short disease duration and minimal prior treatment exposure. Differences between our findings and previous studies reporting stable or increasing GFAP levels under treatment [24,25,26] may therefore be explained by the inclusion of patients with longer disease evolution rather than by true discrepancies in drug effect. These observations support the concept of a therapeutic window of opportunity, during which glial and inflammatory responses remain biologically plastic and amenable to modulation.

The cytokine profile observed in this study further refines this interpretation. The use of ultrasensitive SIMOA technology represents a major strength of the present study. This approach enabled the detection of changes in low-abundance cytokines, that are rarely captured using conventional immunoassays [15]. The ability to quantify immune mediators allows a more refined characterization of immune dynamics in early RRMS. The marked reduction in IFN-γ indicates effective modulation of Th1-associated inflammatory activity [12], which is considered a relatively constant component of MS immunopathology [27]. These findings are consistent with the known mechanism of ozanimod as a selective S1P_1_ receptor modulator that limits the recirculation of activated lymphocytes and reduces their access to the CNS [28]. In parallel, the significant decrease in IL-1β is particularly noteworthy. IL-1β is a hallmark cytokine of the innate immune response and plays a central role in amplifying inflammatory signaling through microglial and astrocytic activation [29,30,31]. The concordant reduction in IL-1β and sGFAP strongly suggests that, in early RRMS, innate immune activation within the CNS remains modifiable and may be indirectly attenuated through effective control of adaptive immune trafficking.

In contrast, IL-17 levels did not decrease significantly when the cohort was analyzed as a whole. However, when patients were stratified according to baseline IL-17 concentrations, a significant reduction emerged in those with values above the cohort median. This finding suggests that ozanimod is capable of modulating Th17-associated inflammation when this pathway is biologically active. The absence of a significant effect in the overall analysis likely reflects the characteristics of the study population, which predominantly comprised patients with low inflammatory burden and limited Th17 activation at baseline. These results highlight the importance of baseline immune context when interpreting cytokine biomarkers and support a more individualized, stratified approach to immunological assessment in MS.

The profile of patients included in this study is central to the interpretation of these results. This was not heterogeneous; in this study all participants had a short disease duration, and only a minority received prior treatment with other DMT. Although patients with low-to-moderate disease activity are often considered to have a favorable prognosis [31,32], sensitive biomarkers such as sNfL and sGFAP reveal that subclinical neuroaxonal and glial injury may already be present at these early stages. In this context, the observed biomarker reductions suggest that early initiation of ozanimod may help intercept pathogenic processes before they evolve into more autonomous and less controllable forms of inflammation and neurodegeneration.

Beyond peripheral immune modulation, the present findings are also compatible with a contribution of central S1P receptor signaling. S1P_1_ and S1P_5_ receptors are expressed not only on lymphocytes but also on CNS-resident cells, including astrocytes and oligodendrocytes [33]. Modulation of S1P_1_ has been implicated in the regulation of astrocytic reactivity and inflammatory signaling [34], while S1P_5_ plays a role in oligodendrocyte survival, myelin maintenance, and resilience to inflammatory stress [35]. Although the present study cannot disentangle peripheral from central effects, the coordinated reduction in sNfL and sGFAP is biologically consistent with an integrated mechanism involving both reduced immune trafficking and modulation of CNS glial biology.

Within the S1P modulator class, differences in receptor selectivity may be relevant to these observations. Ozanimod selectively targets S1P_1_ and S1P_5_, allowing effective control of immune cell egress while potentially engaging CNS-relevant pathways and maintaining a favorable safety profile [36]. Such selective receptor engagement may be particularly advantageous when treatment is initiated early, at a stage when inflammatory and glial processes remain biologically plastic and responsive to modulation.

In summary, this real-world study demonstrates that ozanimod treatment in patients with low-to-moderate activity RRMS is associated with coordinated changes in biomarkers of neuroaxonal damage, astroglial activation, and immune function. The data support a model in which early, selective S1P_1_/S1P_5_ modulation attenuates Th1-driven inflammation, conditionally modulates Th17 activity in patients with higher baseline activation, and influences innate immune pathways, contributing to the preservation of CNS tissue integrity. These findings provide a strong biological rationale for the observation that the effects of ozanimod are more readily detectable when treatment is initiated within a temporal window in which inflammatory and glial responses remain modifiable.

Importantly, as the present study was conducted in a population characterized by low-to-moderate disease activity, further studies including patients with higher disease activity will be required to determine whether the biomarker effects observed here are also evident in more active RRMS populations. In this context, the integration of highly sensitive serum biomarkers may help identify patients who are most likely to benefit from early intervention, thereby informing personalized treatment strategies aimed at maximizing long-term disease control in RRMS. These findings will be further validated in future multicenter studies.

## 4. Materials and Methods

### 4.1. Study Design

This was an observational, multicenter, ambispective study including RRMS patients under ozanimod treatment in routine clinical practice aligning with the Strengthening the Reporting of Observational Studies in Epidemiology (STROBE) statement [37], conducted between June 2023 and December 2025 in 19 Spanish hospitals by neurologists which provided serum samples of a total of 38 centers involved in the AppreZiate study (NCT05811416).

Patients were eligible for inclusion if they met all of the following criteria: age ≥ 18 years at treatment start; a confirmed diagnosis of RRMS according to the 2017 revised McDonald criteria [38]; low-to-moderate disease activity, defined as fewer than 2 relapses in the year prior to initiating ozanimod treatment [39,40]; and initiation of ozanimod for the first time within 3 months (±2 weeks) before study inclusion, in accordance with the EU Summary of Product Characteristics (SmPC) [41] and/or the Spanish Therapeutic Positioning Report (TPR) [42]. Patients were excluded if they had prior exposure to ozanimod, had received more than one DMT before starting ozanimod, or had initiated ozanimod treatment within the context of a clinical trial. Written informed consent was obtained from all patients meeting the eligibility criteria prior to enrollment.

**Figure 8:**
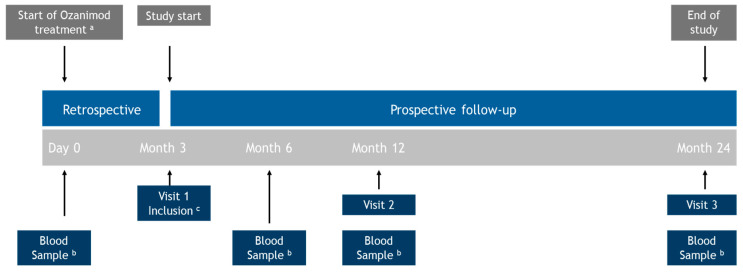
Design of AppreZiate study. ^a^ The day of starting treatment with ozanimod will be considered as Day 0. ^b^ If available, use leftovers from routine blood analysis, stored under routine procedures. ^c^ Visit 1 (inclusion) will take place 3 months ± 2 weeks after Day 0. During visit 1, informed consent will be obtained.

Study design is shown in Figure 8. It was an ambispective study including retrospective 3-month data and 9 months for prospective follow-up. It received approval from the ethics committee of Hospital Universitario Ramón y Cajal.

### 4.2. Data Collection

Retrospective data were collected from patients’ medical records, while prospective data were obtained from blood sample results analyzed at the central laboratory (Hospital Ramón y Cajal, Madrid, Spain). Patients were prospectively followed at each participating hospital through biannual visits, with additional assessments conducted in the event of clinical relapses. Clinical data were recorded in a centralized database by the investigator responsible at each center and subsequently submitted to the study coordinator.

Data collection followed two approaches according to patient status at study inclusion. Retrospective data were collected from all enrolled patients, spanning from baseline to the index date (i.e., 3 months after treatment initiation [±2 weeks]), or until ozanimod discontinuation in patients who discontinued treatment before reaching 3 months. Prospective data were collected exclusively from patients who were actively receiving ozanimod at the time of study inclusion, from treatment initiation until 12 months after ozanimod start or discontinuation, whichever occurred first.

Baseline demographic, clinical, and radiological variables were collected. Baseline was defined as the date of ozanimod treatment initiation. The outcomes assessed included biomarkers of axonal damage and astrocyte activation, measured through sNfL and sGFAP levels, as well as immune function, evaluated through IFN-γ, IL-17, IL-1β, IL-6, and IL-10 measurements.

#### Neuroimaging Protocol

MRI was performed on 1.5 T scanners (Ingenia or Achieva; Philips Healthcare, Best, The Netherlands) using standard clinical protocols, including axial T1-weighted and T2-FLAIR sequences for brain lesion assessment.

Neuroimaging biomarkers were assessed using Neurocloud-VOL, an FDA-cleared CE-marked artificial intelligence (AI) tool for analysis of MRI assisted by QuBiotech (QuBiotech, Coruña, Spain). Inflammatory lesions were automatically identified on co-registered T1-weighted and T2-FLAIR images using a validated lesion detection pipeline.

For the purposes of the present study, only the total number of brain inflammatory lesions at baseline was included in the analysis. All images were anonymized prior to processing and handled according to GDPR-compliant procedures.

### 4.3. Serum Biomarkers and Cytokine Quantification

Patient blood samples were collected and serum was aliquoted and stored at −80 °C until analysis. sNfL, sGFAP, IFN-γ, IL-17, IL-6, IL-10 and IL-1β were measured by single-molecule array (SIMOA) technique (Quanterix, Billerica, MA, USA) in a HDX device (Quanterix, Lexington, MA, USA) following the manufacturer’s instructions. For measuring sNfL and sGFAP, we used the Neurology 2-Plex B Advantage PLUS Kit (Quanterix, Billerica, MA, USA; cat#104670), according to the manufacturer’s instructions. Levels of cytokines were measured in serum samples using commercially available immunoassay kits (Cytokine 4-Plex A (C4PA) Advantage PLUS, cat#104979, for IL-6, IL-10 and IL-1β; IFN-γ Advantage Kit, cat#103337, for IFN-γ; and IL-17A Advantage PLUS, cat#104428, for IL-17 (Quanterix, Billerica, MA, USA)), run on the fully automated ultrasensitive SIMOA HD-X Analyzer (Quanterix). Samples were run in single in accordance with manufacturers’ instructions with appropriate standards and internal controls run in duplicate. Both the mean intra-assay coefficient of variation of duplicates and the mean inter-assay coefficient of variation were <10% for all assays. Investigators involved in the assessment of different serum samples were blind to clinical data.

### 4.4. Definitions

Disability was evaluated through the Expanded Disability Status Scale (EDSS) score [43], a 10-point scale developed from the combination of impairments in eight different domains, where 0 indicates normal neurological examination and 10 indicates death due to MS [43,44]. Relapses were defined as subacute new or worsening clinical symptoms lasting for at least 24 h and separated from a previous attack by a minimum of 30 days.

### 4.5. Statistical Analysis

Continuous variables were summarized using mean, median, standard deviation (SD), and interquartile range (IQR). Categorical variables were described as absolute frequencies and percentages (n, %).

For comparisons of repeated measures of the soluble markers we used nonparametric ANOVA tests for paired data (Friedman test) with post hoc analysis by Dunn’s multiple comparison tests.

All statistical tests were two-sided, with a significance level set at *p* < 0.05. Analyses were conducted on observed data only, with no imputation performed for missing values. All analyses were performed using GraphPad Prism (version 9.5).

## 5. Conclusions

Ozanimod treatment in patients with low-to-moderate activity RRMS was associated with coordinated reductions in serum biomarkers of neuroaxonal damage, astroglial activation, and selected inflammatory pathways over 12 months in real-world clinical practice. The concomitant reductions in sNfL, sGFAP, IFN-γ, and IL-1β are consistent with potential biological effects involving preferential Th1 and innate immune modulation, conditional effects on Th17 activity, depending on baseline immune status, and potential central effects mediated by selective S1P_1_/S1P_5_ modulation. Importantly, these findings highlight the relevance of early disease stage, in which inflammatory and glial responses remain biologically plastic and amenable to intervention. The results reinforce the rationale for early ozanimod initiation within the therapeutic window of opportunity and highlight the value of integrating serum biomarkers to monitor treatment response, ultimately supporting personalized treatment strategies aimed at long-term disease control in RRMS.

## Figures and Tables

**Table 1:** Demographic data and baseline characteristics of patient population.

Values at Start of Ozanimod Treatment	Cohort (n = 55)
**Age, median (IQR)**	45.0 (39.0–51.0)
**Gender, n (%)**	Female = 35 (63.6%) Male = 20 (36.4%)
**Age at symptoms onset, median (IQR)**	39.5 (33.0–46.00)
**Age at diagnosis, median (IQR)**	42 (35.0–49.0)
**Untreated patients, n (%)** **Patients previously treated with a DMT, n (%)**	50 (90.9%) 5 (9.1%)
**Number of relapses in the previous year per patient**	
Mean (SD)	0.60 (0.50)
Median (IQR)	1.0 (0.00–1.00)
**EDSS score**	
Mean (SD)	1.60 (1.01)
Median (IQR)	1.50 (1.00–2.00)
**Total number of lesions**	
Mean (SD)	37.82 (31.34)
Median (IQR)	25.00 (17.00–54.00)

DMT: Disease-modifying therapy; EDSS: Expanded Disability Status Scale; IQR: interquartile range; SD: standard deviation.

## Data Availability

The data presented in this study are not publicly available due to privacy and ethical restrictions related to individual patient data.

## Abbreviations

The following abbreviations are used in this manuscript:

CNS	Central Nervous System
DMT	Disease-Modifying Treatment
EDSS	Expanded Disability Status Scale
IFN	Interferon
IL	Interleukin
IQR	Interquartile Range
MRI	Magnetic Resonance Imaging
MS	Multiple Sclerosis
RRMS	Relapsing–Remitting Multiple Sclerosis
SD	Standard Deviation
sGFAP	Serum Glial Fibrillary Acidic Protein
SIMOA	Single-Molecule Array
SmPC	Summary of Product Characteristics
sNfL	Serum Neurofilament Light Chain
STROBE	Strengthening the Reporting of Observational Studies in Epidemiology
S1P	Sphingosine 1-Phosphate
Th	T Helper
TPR	Therapeutic Positioning Report
